# *PDYN* rs2281285 Variant Association with Drinking to Avoid Emotional or Somatic Discomfort

**DOI:** 10.1371/journal.pone.0078688

**Published:** 2013-11-06

**Authors:** Ulrich W. Preuss, Stacey J. Winham, Joanna M. Biernacka, Jennifer R. Geske, Georgy Bakalkin, Gabriele Koller, Peter Zill, Michael Soyka, Victor M. Karpyak

**Affiliations:** 1 Department of Psychiatry, Ludwig-Maximilians-University, Munich, Germany; 2 Department of Psychiatry, Martin-Luther-University, Halle-Wittenberg, Germany; 3 Department of Health Sciences Research, Mayo Clinic, Rochester, Minnesota, United States of America; 4 Department of Psychiatry and Psychology, Mayo Clinic, Rochester, Minnesota, United States of America; 5 Division of Biological Research on Drug Dependence, Department of Pharmaceutical Biosciences, Uppsala University, Uppsala, Sweden; 6 Private Hospital Meiringen, Meiringen, Switzerland; St. Luc University Hospital, Belgium

## Abstract

**Introduction:**

One of the proposed psychobiological pathways of craving attributes the desire for drinking in the context of tension, discomfort or unpleasant emotions, to “negative” (or “relief”) craving. The aim of this study was to replicate a previously reported association of the *PDYN* rs2281285 variant with negative craving using a different phenotyping approach.

**Methods:**

The TaqMan® Genotyping Assay was used to genotype the rs2281285 variant in 41**7** German alcohol-dependent subjects. The presence of negative/relief craving was assessed by asking if participants ever ingested alcohol to avoid unwanted emotional or somatic discomfort.

**Results:**

The minor allele of rs2281285 was associated with an increased risk of drinking to avoid/escape unwanted emotional or somatic events (OR = 2.29, 95% CI = 1.08–4.85, p = 0.0298).

**Discussion:**

Despite the use of a different phenotyping approach to the measurement of negative craving, our results confirm the association between negative craving and *PDYN* rs2281285. Genetic markers of negative craving may help to identify subgroups of alcohol-dependent individuals vulnerable to relapse in the context of negative emotions or somatic discomfort, leading to the development of specifically tailored treatment strategies.

## Introduction

Clinical research indicates that negative affect contributes to relapse along with craving and other factors [1], [2]. A previous study [3] proposed a three-pathway psychobiological model of craving. One of the pathways in this model attributes the desire for drinking in the context of tension, discomfort or unpleasant emotions, to “negative” (or “relief”) craving, while the other two pathways relate to obsessive thoughts about drinking (“obsessive” craving) and the desire for rewarding features of alcohol inebriation (positive or “reward”) craving [3]. Previous research also indicated that negative craving may be the most important trigger for relapse (compared to the two other craving pathways), particularly in chronic alcohol-dependent individuals who drink to avoid the unwanted symptoms of withdrawal and mood dysregulation [4], [5], [6].

Several lines of evidence have demonstrated that negative feelings and emotions may be related to the dynorphin (DYN)/ κ-opioid receptor (KOR) system, which may influence the motivational aspects of stress by mediating pro-depressive-like states that involve elements of anhedonia, dysphoria, as well as, pain and aversion in humans and laboratory animals [7], [8], [9], [10], [11]. DYNs, which are posttranslational products of the prodynorphin (*PDYN*) gene, are the endogenous ligands for KOR. Activation of DYN/KOR neurotransmission modulates the activity of dopaminergic and glutamatergic neurons located therein. KOR and DYNs are enriched in brain circuits that control mood, motivation, and stimulus-response (habit) formation and have been implicated in drug-seeking behavior.

Although the function of KOR in alcoholism is not entirely clear [12], multiple lines of evidence suggest κ-opioid system involvement in the development of alcoholism [13]. KOR antagonists could be effective in the treatment of alcoholism [14]. Recently, the effectiveness of nalmefene, a μ-opioid receptor antagonist and partial κ-agonist [12], [15], has been demonstrated in an “as needed” approach to alcoholism treatment [16], [17].

Previous studies found associations between the broad phenotype of alcohol dependence and sequence variation in human *OPRK1* (encoding KOR) and *PDYN* genes [18], [19], [20]. In a study of 816 alcohol dependent subjects and 1248 controls [21], we found a statistically significant relationship between *PDYN* haplotypes containing the rs2281285 variant and negative craving measured by a subscale of the Inventory of Drug Taking Situations (IDTS) [22], [23], [24]. Single SNP associations were also observed between rs2281285 in alcohol dependence (p = 0.0083) and negative craving (p = 0.012). As a replication attempt for the original study, association between rs2281285 and alcohol dependence was examined in a smaller sample of 474 alcohol-dependent subjects and 432 non-alcoholic controls of German descent. Although the odds ratio estimate was consistent with that in the discovery sample, association did not reach statistical significance in the replication sample (OR = 1.18 vs. OR = 1.30; p = 0.22) [21].

In the initial study [21], a replication of the association with rs2281285 and negative craving was not examined, as no IDTS data were available in the replication cohort of German subjects. However, subsequent review of the phenotype data revealed that a majority of German subjects answered the Semi-Structured Interview for the Assessment on the Genetics of Alcoholism (SSAGA) question [25], [26], “Have you ever ingested alcohol to avoid/escape unwanted emotional or somatic discomfort?” Provided that the conceptual meaning of this question is similar to the concept of negative (relief) craving, we hypothesized that this phenotype may also be associated with the *PDYN* rs2281285 variant. The aim of the current study was to test this hypothesis.

## Methods

### Ethics statement

Signed informed consent was obtained from subjects and controls after a complete and extensive description of the study was provided. The Ethics Committee of the Ludwig-Maximilians-University of Munich approved the study.

### Study sample and assessments

Details of the sample acquisition and methods have been described in detail in previous publications. The clinical and genotyping data were provided by a gene bank initiated in Germany in 1998 [27],[28]. Methods of patient assessment and phenotypic characterization were derived from the US COGA project (Collaborative Study on Genetics of Alcoholism) [26], [29]. Subjects were consecutively recruited from an inpatient addiction treatment ward for alcohol-dependent patients. All subjects were of German descent, 18 years and over, met both ICD-10 and DSM-IV criteria for alcohol dependence, and were assessed with the Structured clinical interview according to DSM IV, German version (SCID) [30]. Additional characteristics of alcohol dependence were gathered using the SSAGA and a comprehensive psychiatric examination by one of the authors (UWP or GK). All patients were examined two weeks after admission, and were free of any withdrawal symptoms and psychopharmacological treatment. The occurrence of negative craving was assessed with respective SSAGA-items, where patients were asked if they ever ingested alcohol to avoid unwanted emotional or somatic discomfort, e.g. depressive or anxious mood, sleeplessness, nervousness, tachycardia or somatic pain (yes/no response).

### Genotyping

Genotyping of *PDYN* rs2281285 was described in more detail previously [1], [21]. In brief, the TaqMan® SNP Genotyping Assay (Applied Biosystems, USA) was used to genotype rs2281285 in 915 alcoholic and control subjects. The genotypes showed no deviations from Hardy–Weinberg Equilibrium. As part of quality control, rs2281285 genotyping was duplicated for 81 subjects with 100% concordance. Genotype counts are presented as Table S1.

### Statistical analyses

Analysis was restricted to alcohol-dependent subjects with available data on negative craving. For those subjects, demographic and clinical factors such as age, gender and drinking and mood characteristics were examined for potential confounding associations. Characteristics of alcohol-dependent individuals with and without negative craving were compared using Chi-Square and Wilcoxon rank sum tests.

The minor allele frequencies in alcohol-dependent subjects with and without negative craving were compared assuming an additive genetic model. In the previous study [21], we used a quantitative measure of negative craving (negative subscale of the IDTS) to test for association with SNP variants using linear regression. In the present study, a quantitative measure of negative craving using the IDTS was not available, and negative craving was assessed using the SSAGA question regarding the use of alcohol to escape unwanted emotional events (yes/no). Therefore association was assessed with logistic regression, where negative craving was treated as a binary outcome. The rs2281285 genotype was coded as 0, 1, or 2 to represent the number of copies of the minor allele (i.e. an additive model was assumed), and the logistic regression model was adjusted for age and gender in order to consistently replicate the association observed in the previous publication [21].

In addition, a multivariate logistic regression model was fit including all significant demographic and clinical factors to assess the impact of rs2281285 on negative craving after accounting for other relevant covariates. Because age, age of onset, and duration of dependence are highly correlated, only age of onset was chosen for inclusion to reduce multicollinearity (because it was most strongly associated with negative craving in univariate analysis).

## Results

### Description of clinical sample characteristics and their associations with negative craving

Of the 915 subjects that were genotyped at rs2281285, nine subjects failed genotyping (6 cases and 3 controls), leaving 474 samples from alcoholics and 432 samples from controls available for analyses. Data on negative craving was available for 417 alcoholics and subsequent analyses were restricted to these subjects. Of those, 304 subjects answered “yes” to the SSAGA question if they “ever used alcohol to avoid/escape unwanted emotional or somatic events” and, therefore, were characterized as those with negative craving.

Comparison of demographic and clinical characteristics across groups is presented in Table 1. Age and gender were not significantly associated with negative craving (p = 0.75 and 0.63, respectively). However, patients with negative craving had significantly more severe alcohol dependence than those without, including earlier onset of alcoholism, longer duration of dependence, higher daily consumption prior to admission, and a more frequent history of suicide attempts. Additionally, patients with negative craving had marginally higher rates of delirium tremens and withdrawal seizures history which did not reach a statistically significant level.

**Table 1:** Characteristics of alcohol-dependent individuals with and without negative craving.

	Alcoholics with negative craving (n = 304)	Alcoholics without negative craving (n = 113)	Total Sample (n = 417)	*Chi-Square or Wilcoxon Rank Sum Test P-value*
Gender (males/females)	224/76	87/26	311/102	0.625
Age (years, mean±SD)	43.62±9.9	44.03±10.5	43.5±10.4	0.745
Age of onset (years, mean±SD)	29.96±8.9	32.41±10.4	30.87±9.7	**0.039**
Duration of dependence (years, mean±SD)	13.69±8.8	11.69±8.3	13.00±8.7	**0.040**
Number of positive DSM IV criteria (mean±SD)	5.53±1.2	4.57±1.4	5.27±1.3	**<0.001**
Suicide attempt history (%)	29.1%	19.5%	26.5%	**0.047**
History of DT (%)	21.7%	13.3%	19.4%	0.053
History of WS (%)	21.4%	13.3%	19.2%	0.062
Daily alcohol intake before admission (30d, g/d)	294.72±182.2	219.05±124.6	272.94±170.1	**<0.001**

Abbreviations: DT, delirium tremens; WS, withdrawal seizures; SD, standard deviation of mean

### Associations between negative craving and *PDYN* rs2281285

Table 2 displays the univariate and multivariate logistic regression results of the association between negative craving and rs2281285, age, and gender. Subjects with negative craving have a higher frequency of the *PDYN* rs2281285 minor allele (MAF = 0.18 vs. 0.15 for patients with and without negative craving, respectively). Specifically, the minor allele of rs2281285 was associated with an increased propensity to drink in negative situations, replicating the previous findings [21]. For each copy of the minor allele, the odds of frequently drinking in negative situations is 2.29 times greater (OR = 2.29, p = 0.0298).

**Table 2:** Univariate and multivariate logistic regression results in the replication (German) sample, examining the relationship between negative craving, rs2281285, age, and gender.

	Variable	OR	95% CI	P-value
Univariate Analysis	rs2281285	2.38	(1.18, 4.83)	0.0160
	Gender	0.72	(0.39, 1.34)	0.3024
	Age	1.02	(0.99, 1.05)	0.1824
Multivariate Analysis	rs2281285	2.29	(1.08, 4.85)	0.0298
	Gender	0.78	(0.40, 1.52)	0.4623
	Age	1.02	(0.99, 1.05)	0.1949

## Discussion

Our findings indicate a statistically significant association of the minor allele of the *PDYN* rs2281285 variant with propensity to use alcohol in order to avoid unwanted emotional or somatic discomfort assessed based on a yes/no question included as part of a structured interview (SSAGA), after adjusting for age and gender. These findings are in agreement with our previous finding, which demonstrated that the minor allele of rs2281285 was also associated with increased propensity to drink in negative emotional states measured with the quantitative IDTS scale (β = 6.96, p = 0.012) [21]. Both, the propensity to use alcohol in order to avoid unwanted emotional or somatic discomfort, as well as, the propensity to drink in negative emotional states are conceptualized as negative or “relief” craving, elicited through negative emotions, history of alcohol withdrawal and attributed to an imbalance between glutamate and gamma-amino-butyric acid neurotransmission in the brain [3]. Yet, results presented here, along with the results of our previous study indicate statistically significant positive association of the minor allele of *PDYN* rs2281285 with both phenotypes, which supports the consistency of the finding.

This result also supports involvement of the DYN/KOR system in mechanisms controlling mood and motivation processes involved in alcohol dependence. As reported in the previous publication [21], the association of the *PDYN* rs2281285 variant with the broader phenotype of alcohol dependence could not be found in the validation sample. Yet, in the current analysis, the association between the *PDYN* variant and negative craving was detected within the same sample of alcoholics. This finding indicates the need for further investigation of the genetic factors associated with negative craving and the broadly defined phenotype of alcohol dependence. Moreover, future research is needed to investigate the role of *PDYN* variation and negative craving in alcohol withdrawal and comorbid substance induced and non-substance induced mood problems in alcoholics.

Our data also indicate that more severe clinical manifestations of alcoholism, including length, number of symptoms, higher alcohol consumption and potentially higher frequency of DT and WS are present in alcohol-dependent subjects exhibiting negative craving (Table 1). This is consistent with previous research, which demonstrated that negative craving is associated with a more severe course of alcohol dependence and may be a significant risk factor for relapse [3], [24]. This type of association discovered in cross sectional studies does not answer the question if negative craving is a consequence or a predisposing factor to development of a severe and complicated course of alcohol dependence. However, association of negative craving with the *PDYN* rs2281285 variant discovered in our previous study and replicated here favors the possibility that genetic factors acting alone or in combination with environmental factors (e.g. heavy alcohol consumption) may predispose to negative craving and a more severe course of alcoholism. Prospective studies investigating the sequential relationships between negative craving and phenotypes reflecting severity of alcoholism are necessary to further clarify this possibility. This direction of research, allowing characterization of genetic variants defining subgroups of alcohol-dependent individuals, in this case with and without negative craving, may facilitate future tailored pharmacological and psychotherapeutic treatment strategies for these individuals. As with other alcohol-use-related phenotypes, it is expected that many more than one genetic variant may contribute to this phenotypic presentation. Therefore, further search for genetic variations associated with negative craving is necessary. It is reasonable to focus this search on sequence variation in genes encoding proteins involved in DYN/KOR neurotransmission as well as relevant regulatory regions, which may be located in other parts of the genome.

It is also important to uncover the functional mechanisms underlying the described association between negative craving and the *PDYN* rs2281285 variant. This SNP has been described as a tag SNP for a haplotype block within the *PDYN* gene (across exons and introns 1 and 2) [20]. Yet, it is located in an area that may be involved in the regulation of transcription initiation from multiple sites, giving rise to one of the *PDYN* transcripts [31]. Moreover, the low risk rs2281285 major allele resides within the sequence that allows DNA-binding for several transcription factors, whereas the high risk rs2281285 minor allele destroys this sequence [21]. Therefore, more research is necessary to determine if the *PDYN* rs2281285 variant has functional importance or represents a marker for another functional variant(s).

The results of our study need to be considered in the context of the following limitations. First, while the initial study employed a questionnaire to assess negative craving, the current study obtained information on this phenotype using items of a semi-structured interview. The units of the effect size measures used in the original study and in this replication study are not directly comparable. However, changes in the odds ratio indicate the same direction of association between negative craving and the minor allele of the *PDYN* rs228125 variant, which was statistically significant despite the difference in assessment methods. Second, the samples investigated in both studies included alcohol-dependent individuals involved in treatment, which may be associated with a more severe course of disease. Thus, the results may not be generalizable to other sub-groups of alcoholics with a potentially less severe course of disease. Finally, although the current sample of alcohol-dependent individuals with and without negative craving is larger than the sample with negative craving data analyzed in the initial study (N = 196), it is still relatively small. Validation in a larger sample is needed to assess if generalization to other sub-populations of alcohol dependent subjects is appropriate. Nevertheless, significant evidence of association with negative craving was observed here, replicating the earlier results.

In conclusion, negative craving is an important characteristic in alcohol-dependent individuals that may be linked to genetic variation in the endogenous opioid system and, specifically, the *PDYN* gene. This study confirmed a previous finding that rs2281285 is related to negative craving, although it was measured with a different assessment as compared to the original study. This result supports continuous investigation of the DYN/KOR system as potential pharmacological target for treatment of negative craving and relapse prevention in alcohol-dependent individuals. It also indicates that *PDYN* variants may be among potential pharmacogenomic predictors of such treatment.

## Supporting Information

Table S1 Genotype and allele counts of rs2281285 by presence/absence of the negative craving phenotype.
(DOCX)
