# Supplementary material for: PDYN rs2281285 Variant Association with Drinking to Avoid Emotional or Somatic Discomfort
Source: PLoS One. 2013 Nov 6;8(11):e78688. doi: 10.1371/journal.pone.0078688 (PMC3819371; doi:10.1371/journal.pone.0078688)
Supplement: Table S1 — Genotype and allele counts of rs2281285 by presence/absence of the negative craving phenotype. (DOCX) [file pone.0078688.s001.docx]

**Supporting Information**

***PDYN* rs2281285 Variant Association with Drinking to Avoid Emotional or Somatic Discomfort**

**Table S1: Genotype and allele counts of rs2281285 by presence/absence of the negative craving phenotype**

| **rs2281285** | **TT** | **TC** | **CC** | **T** | **C** |
| --- | --- | --- | --- | --- | --- |
| **Alcoholics with negative craving** | n=203 (66.7%) | n=92 (30.3%) | n=9 (3.0%) | 81.9% | 18.1% |
| **Alcoholics without negative craving** | n=82 (72.6%) | n=28 (24.8%) | n=3 (2.6%) | 85.0% | 15.0% |
